# Decolourisation Capabilities of Ligninolytic Enzymes Produced by* Marasmius cladophyllus* UMAS MS8 on Remazol Brilliant Blue R and Other Azo Dyes

**DOI:** 10.1155/2017/1325754

**Published:** 2017-01-12

**Authors:** Ngieng Ngui Sing, Ahmad Husaini, Azham Zulkharnain, Hairul Azman Roslan

**Affiliations:** Department of Molecular Biology, Faculty of Resource Science and Technology, Universiti Malaysia Sarawak, 94300 Kota Samarahan, Sarawak, Malaysia

## Abstract

*Marasmius cladophyllus* was examined for its ability to degradatively decolourise the recalcitrant dye Remazol Brilliant Blue R (RBBR) and screened for the production of ligninolytic enzymes using specific substrates. Monitoring dye decolourisation by the decrease in absorbance ratio of *A*_592_/*A*_500_ shows that the decolourisation of RBBR dye was associated with the dye degradation.* Marasmius cladophyllus* produces laccase and lignin peroxidase in glucose minimal liquid medium containing RBBR. Both enzyme activities were increased, with laccase activity recorded 70 times higher reaching up to 390 U L^−1^ on day 12. Further in vitro RBBR dye decolourisation using the culture medium shows that laccase activity was correlated with the dye decolourisation. Fresh RBBR dye continuously supplemented into the decolourised culture medium was further decolourised much faster in the subsequent round of the RBBR dye decolourisation. In vitro dye decolourisation using the crude laccase not only decolourised 76% of RBBR dye in just 19 hours but also decolourised 54% of Orange G and 33% of Congo red at the same period of time without the use of any exogenous mediator. This rapid dye decolourisation ability of the enzymes produced by* M. cladophyllus* thus suggested its possible application in the bioremediation of dye containing wastewater.

## 1. Introduction

Synthetic dyes are widely used throughout the world for various purposes particularly for textile dyeing. Each year, more than 80,000 tons of reactive dyes are produced and consumed for textile dyeing. Given their beneficial characteristic of bright colour, water-fast, and simple application techniques with low energy consumption, reactive dyes have been used extensively to dye more than half of the global production of cotton [1]. However in the process of textile dyeing, 50–60% of these water soluble dyes do not bind to the fiber and are lost into the effluent. In addition to the high volume of water required, each year textile mills generate and discharge billions of liters of wastewater effluent full of colours, salts, and organic chemicals harmful to the environment [1, 2].

Remazol Brilliant Blue R (RBBR) dye, also known as Reactive Blue 19, is a typical reactive dye used in the textile industry for dyeing cellulosic fibers. It is an anthraquinone-based vinylsulphone dye frequently used as the starting material in the production of polymeric dyes. RBBR dye however has relatively low fixation efficiency (75–80%) on cellulose due to the competition between the formation of reactive vinylsulphone and 2-hydroxyethylsulfone in which the latter does not attach to cellulose fiber [3, 4]. When released without adequate treatment, RBBR dye can persist for a long period of time in the environment due to the fused aromatic ring structures of the dye which are difficult to degrade. At a temperature of 25°C and under normal pH of 7, RBBR dye has been reported to possess a half-life as long as 46 years [3]. Moreover, RBBR is an anthracene derivative and represents an important class of toxic and recalcitrant organopollutants [5]. Dye containing wastewater therefore must be treated before their discharge into the environment.

In recent years, interest in using microorganisms to treat dye containing wastewater has increased not only because of its cost effectiveness and ease of application but also because of the ecofriendliness of this treatment method that generates less toxic chemical sludge [6]. The interest in using biological treatment method as compared to conventional physicochemical method can be seen with the growing number of research and review papers that have been putting forth evidences on the feasibility of using fungi particularly white rot fungi for dye decolourisation [7–10]. White rot fungi, a group of lignin degrading basidiomycetes, have been shown to be able to degrade a wide range of organic pollutants including synthetic dyes due to the nonspecific and nonstereoselective nature of the ligninolytic enzymes system produced by these fungi. The ligninolytic enzymes of white rot fungi, consisting of lignin peroxidase, manganese peroxidase, and laccase, have all been reported to be involved in RBBR decolourisation [7].

We have previously reported on the successful isolation of an endophytic fungus* Marasmius cladophyllus* UMAS MS8 that was able to decolourise several synthetic dyes including RBBR on both solid and liquid mediums [11]. Detailed study on the dye decolourisation potential of* Marasmius *sp. such as isolate UMAS MS8 thus far has rarely been reported. Possible reasons could be because of the low lignin modifying enzyme production and low dye decolourisation ability that was reported previously for several* Marasmius* sp. [12, 13].* Marasmius* sp. particularly* Marasmius quercophilus* however has been reported to produce laccase and attempts have also been made to purify the enzyme for the degradation of xenobiotic aromatic compounds [14–16].* Marasmius scorodonius *has also been reported to produce novel peroxidase capable of degrading beta-carotene [17]. Recently, a report of an unusual one copper laccase capable of decolourising several dyes including RBBR was also produced by* Marasmius* sp. [18]. In this study, we investigated the production of ligninolytic enzymes by an endophyte* M. cladophyllus* during RBBR dye decolourisation and the possibility of these ligninolytic enzymes being differentially expressed during the decolourisation process. Here, we also reported on a rare attempt on reusing enzymes harvested from RBBR decolourised medium for the decolourisation of the most widely used dye, the azo type dyes.

## 2. Materials and Methods

### 2.1. Fungus and Culture Maintenance

*Marasmius cladophyllus* UMAS MS8 was an endophytic fungus that was previously isolated from* Melastoma malabathricum*. The fungal isolate (referred to as isolate UMAS MS8 from here onward) was maintained by serial transfer on malt extract agar (MEA) and kept on MEA agar slants at 4°C. Fungal stock was also prepared in 20% (v/v) glycerol and kept at −20°C.

### 2.2. RBBR Dye Decolourisation in Liquid Media

Isolate UMAS MS8 was tested for RBBR dye decolourisation in glucose minimal (GM) liquid medium. The GM liquid medium contained (per liter) 1 g of K_2_HPO_4_, 0.01 g of ZnSO_4_·7H_2_O, 0.005 g of CuSO_4_·5H_2_O, 0.5 g of MgSO_4_·7H_2_O, 0.01 g of FeSO_4_·7H_2_O, 0.5 g of KCl, 10 g of glucose, and 3 g of NaNO_3_ as the sole source of nitrogen. The pH of the liquid medium was adjusted to 5.5 before autoclaving at 121°C for 15 minutes. Remazol Brilliant Blue R (RBBR) dye was added to 20 mL of the autoclaved GM liquid medium in 100 mL Erlenmeyer flask to a final concentration of 0.2 g L^−1^. Each flask was inoculated with two pieces of agar plugs (5 mm^2^ in diameter) taken from a 7-day-old MEA grown fungal culture and incubated in the dark at room temperature under static condition. Flask with dye but with no fungal inoculum was used as abiotic control. Control flask with no dye but inoculated with fungus was also prepared. Each culture condition was prepared in triplicate and incubated for 15 days with sampling at a 3-day interval. On sampling, the whole cultures were used and centrifuged at 6000 rpm for 10 min, to separate the fungal mycelium from the culture medium. Degradative dye decolourisation by the fungus was measured by monitoring the absorbance of RBBR dye in the culture medium at its maximum absorption wavelength (592 nm) and also at 500 nm using a UV-vis spectrophotometer (UV mini 1240, Shimadzu). Possible dye adsorption onto fungal mycelium was determined by the extraction with methanol for spectrophotometric examination. Remazol Brilliant Blue R (RBBR) dye decolourisation was reported in the form of absorbance ratio* A*_592_/*A*_500_ [19].

### 2.3. Ligninolytic Enzyme Assay

The production of the three major ligninolytic enzymes, namely, lignin peroxidase (LiP), manganese peroxidase (MnP), and laccase (Lac), by isolate UMAS MS8 during RBBR dye decolourisation was measured from an aliquot of the culture medium harvested at a 3-day interval. Ligninolytic enzyme activity in culture broth from control flasks with no RBBR dye but inoculated with isolate MS8 was also measured, to study the possibility of differential ligninolytic enzyme production by the fungus in the present and absence of RBBR dye. Lignin peroxidase (LiP) activity was measured by monitoring the oxidation of veratryl alcohol to veratraldehyde at 310 nm (molar absorptivity, *ε*_310_ = 9300 M^−1 ^cm^−1^) in 50 mM sodium tartrate buffer (pH 2.5), in the presence of 0.4 mM H_2_O_2_. Activity of MnP was estimated by monitoring the formation of Mn^3+^ tartrate complex during the oxidation of Mn^2+^ at 238 nm (molar absorptivity, *ε*_238_ = 6500 M^−1 ^cm^−1^). The reaction mixture contained 100 mM sodium tartrate buffer (pH 5), 0.1 mM MnSO_4_, and 0.1 mM of H_2_O_2_. Laccase (Lac) activity was measured based on the oxidation of the substrate 2,2′-azinobis(3-ethylbenzothiazoline-6-sulphonic acid) diammonium salt (ABTS) at 420 nm (*ε*_420_ = 36000 M^−1 ^cm^−1^) in 0.1 M sodium acetate buffer (pH 5). One unit of enzyme activity was defined as the amount of enzyme required to oxidize 1 *μ*mol of substrate per min [20–22].

### 2.4. In Vitro RBBR Dye Decolourisation Using Fungal Crude Enzyme

The culture medium harvested at a 3-day interval was used as crude enzyme for in vitro RBBR dye decolourisation carried out in test tubes. The reaction mixture contained 2 mL of the crude enzyme and 0.2 g L^−1^ of RBBR dye in a final volume of 2.02 mL. After an hour of incubation at room temperature, dye decolourisation was measured by monitoring the decrease in absorbance maximum of RBBR dye (592 nm) using UV-vis spectrophotometer, in parallel with control samples prepared using heat inactivated crude enzyme. Crude enzyme prepared from culture medium inoculated with isolate UMAS MS8 but without dye was also tested for in vitro RBBR dye decolourisation. Percentage of dye decolourisation was calculated according to the following equation:

(1) $$\begin{array}{c} \text{Percentage of decolourisation }\left(\;\%\;\right)=\frac{A_{c}-A_{s}}{A_{c}}\times 100, \end{array}$$

where *A*_*c*_ is the absorbance at the maximum absorption wavelength of dye in the heat inactivated crude enzyme at time *t* and *A*_*s*_ is the absorbance at the maximum absorption wavelength of dye in the sample crude enzyme at time *t* [23].

### 2.5. Successive Dye Addition and Decolourisation

Isolate UMAS MS8 was cultured similarly as mentioned earlier in GM liquid medium containing 0.2 g L^−1^ of RBBR dye. When dye decolourisation reached more than 80%, fresh RBBR dye was supplemented to the culture medium to a final concentration of 0.2 g L^−1^ for another cycle of dye decolourisation. Samples were taken out from the culture medium at appropriate time intervals and analysed for dye decolourisation by monitoring the decrease in absorbance maximum of RBBR dye (592 nm) using a UV-vis spectrophotometer. Fresh RBBR dye was supplemented to the culture medium two times [24].

### 2.6. In Vitro Azo Dye Decolourisation Using Ammonium Sulphate Precipitated Protein

The protein content of day 6 crude enzyme obtained from RBBR decolourised culture medium was isolated by stepwise precipitation using different amount of solid ammonium sulphate (10%–100%, with 10% increment). Protein precipitated from each fraction after resuspended in 0.1 M sodium acetate buffer (pH 5) was tested for in vitro RBBR dye decolourisation and ligninolytic enzyme activity, via the protocol as mentioned earlier. Protein precipitates from 10 to 50% were all discarded as no RBBR dye decolourisation and no ligninolytic enzyme activity were observed. For protein precipitated at 60 to 100% saturation, only laccase activity was detected and all protein precipitates were able to decolourise RBBR dye. The ability of these laccase containing precipitates to decolourise different azo type dyes, namely, Congo red and Orange G, was tested in vitro. The 2.02 mL reaction mixture contained 0.1 M sodium acetate buffer (pH 5), 0.9 U laccase activity, and 0.2 g L^−1^ of the azo dye. Similar test was also set up for RBBR dye and control using heat inactivated enzyme. Dye decolourisation after 19 hours was monitored by scanning the spectrum between 380 and 800 nm using a UV-vis spectrophotometer after diluting the reaction mixture to less than 1.5 absorbance units at the maximum absorption wavelength of the tested dye [25].

### 2.7. Zymograms of Native-PAGE

Zymogram for detection of laccase isoform in the RBBR dye decolourised culture medium and the ammonium sulphate protein precipitate was performed under nondenaturing conditions with 12% polyacrylamide gel. After electrophoresis, laccase activity was visualised by staining with 1 mM ABTS solution in sodium acetate buffer (0.1 M, pH 5.0) for the formation of green laccase band [22].

## 3. Results and Discussion

### 3.1. RBBR Dye Decolourisation in Liquid Medium

**Figure 1:**
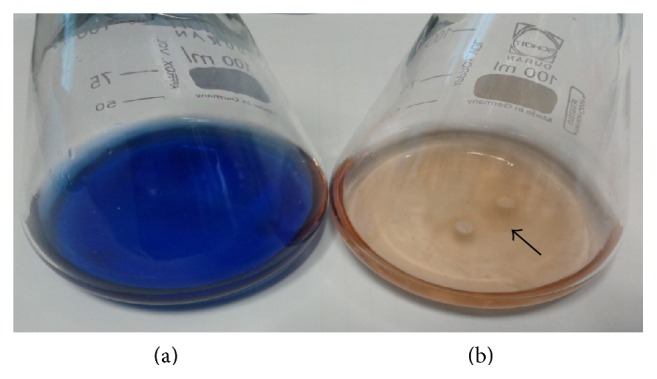
Decolourisation of RBBR dye (0.2 g L^−1^) by* M. cladophyllus* UMAS MS8 in glucose minimal medium. (a) Abiotic control with no fungal inoculum. (b) Decolourised RBBR dye after 15 days by* M. cladophyllus* with no visible dye absorbed onto the fungal mycelium (arrow).

**Figure 2:**
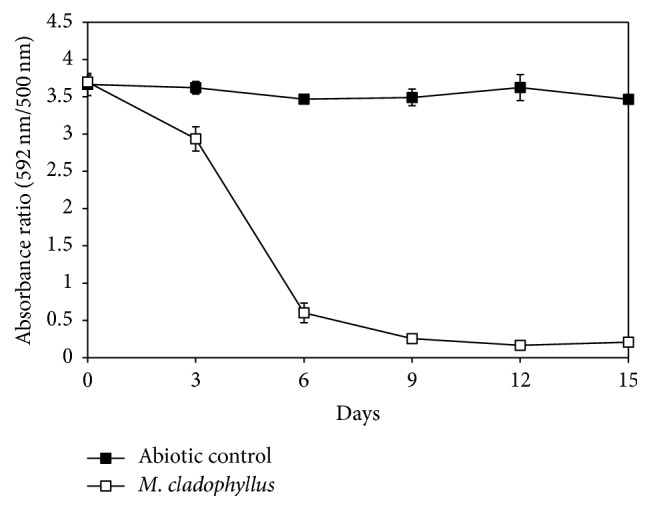
Absorbance ratio showing degradation of RBBR dye (0.2 g L^−1^) in glucose minimal medium by* M. cladophyllus* UMAS MS8 within a period of 15 days.

The fungal isolate MS8 was able to decolourise RBBR dye in the GM liquid medium (Figure 1) as can be seen with the disappearance of the blue coloured dye in flask treated with the fungus (Figure 1(b)). To further distinguish whether RBBR decolourisation by isolate MS8 was by means of degradative colour removal or by adsorption, dye decolourisation was reported in absorbance ratio of* A*_592_/*A*_500_ and possible dye adsorption onto fungal mycelium was also quantified after methanol extraction. Degradative decolourisation of RBBR causes the absorbance ratio to decrease due to the decrease in absorbance at the maximum absorption wavelength (592 nm). Meanwhile, decolourisation by adsorption will cause the ratio to remain fairly constant as there are similar decreases in absorbance across the whole spectrum [26]. From the results obtained, it is shown that the absorbance ratio in flask inoculated with isolate UMAS MS8 decreases while the absorbance ratio in the abiotic control flask remained almost constant throughout the 15 days of incubation (Figure 2). This therefore indicated the degradative decolourisation of RBBR dye by isolate MS8. In the flask inoculated with isolate MS8, the absorbance ratio decreases from 3.7 on day 0 to 2.9 on day 3 and further decreased to 0.6 on day 6 before reaching 0.3 on day 9. In a period of 6 days, isolate MS8 decolourised more than 82% of the RBBR dye initially added (0.2 g L^−1^). On day 6, when most of the dye has been removed, less than 3% of the dye was found on the fungal biomass. These results further shows that the high percentage of dye decolourisation by the fungus was mainly via degradation or metabolism of the dye and not by adsorption onto fungal mycelium [24, 27].

### 3.2. Production of Ligninolytic Enzyme

**Figure 3:**
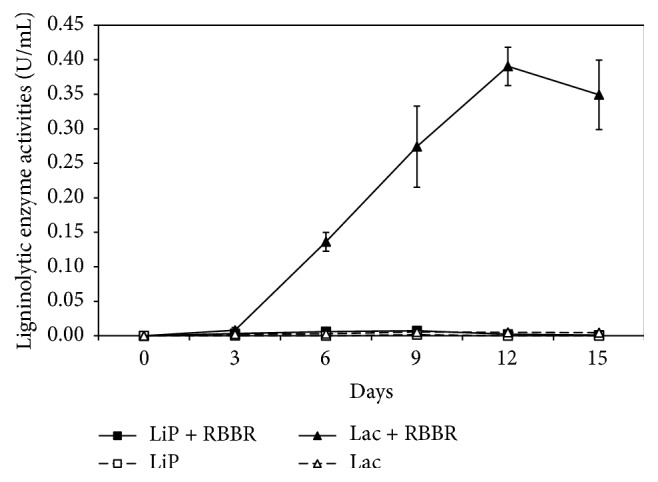
Lignin peroxidase and laccase activities produced by* M. cladophyllus* in 15 days in culture medium with and without RBBR dye.

In the presence and absence of RBBR dye, isolate UMAS MS8 was found to produce both Lac and LiP but no detectable amount of MnP activity (Figure 3). In liquid medium containing RBBR dye, Lac was found to be the dominant enzyme produced while only minute amount of LiP was detected. Both enzyme activities increased from day 0 and reached a maximum on day 9 for LiP (7 U L^−1^) and on day 12 for Lac (390 U L^−1^). When compared with the degradative decolourisation of RBBR dye by isolate MS8, the increase in enzyme activities coincides with the rapid decrease of RBBR dye from day 0 to day 9 (Figure 2). This therefore suggested the possible involvement of both enzymes in the decolourisation of RBBR dye.

On the other hand, in the liquid medium without RBBR dye, isolate MS8 produced only a small amount of both Lac and LiP (Figure 3). Lac activity increased from day 0 to day 9 reaching a maximum of 6 U L^−1^ within 15 days of incubation, while LiP activity was only detected on day 9 (2 U L^−1^). In the presence of RBBR dye, enzyme activity of both the LiP and Lac was constantly higher as compared to activity in liquid medium without the dye. On day 12, Lac activity in liquid medium containing RBBR was detected to be 70-fold higher than Lac activity in medium without the dye. In addition, LiP activity on day 9 was also recorded three times higher in liquid medium containing RBBR. The results showed that the presence of RBBR dye induced the production of both the LiP and Lac enzymes by isolate MS8. The increase in ligninolytic enzyme activity, particularly Lac, was most probably needed for the decolourisation of RBBR dye by isolate MS8. Similar results have also been reported for a white rot fungus,* Ischnoderma resinosum*, during the decolourisation of synthetic dyes [28]. Orange G and RBBR dye have increased the Lac activity of* I. resinosum* 20- and 10-fold, respectively, after 20 days of dye decolourisation. Malachite green has also brought about a 5-fold increase in Lac activity of the fungus and the increase in enzyme activity was positively correlated with the efficiency of dye decolourisation [29].

### 3.3. In Vitro RBBR Dye Decolourisation

**Figure 4:**
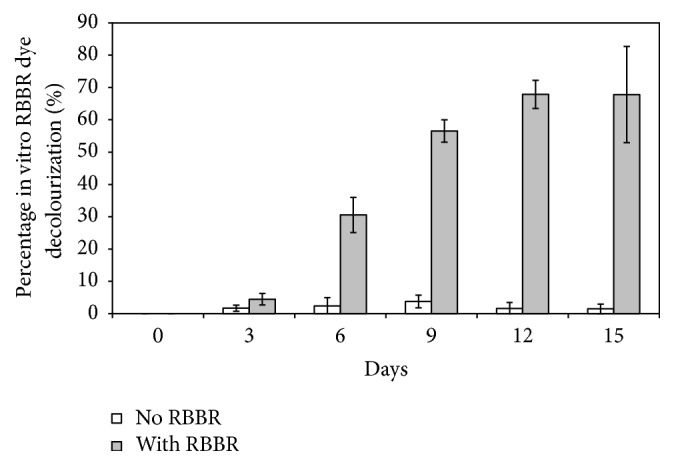
In vitro RBBR dye decolourisation using crude enzyme harvested from culture medium containing RBBR dye and culture medium without RBBR dye within a period of 15 days.

Following the increased enzyme activities induced by RBBR dye, in vitro decolourisation of RBBR dye was conducted using the culture medium containing enzymes. Interestingly, the result showed that the harvested culture medium was able to decolourise RBBR dye in vitro and the higher the ligninolytic enzymes content in the culture medium, the higher the percentage of RBBR dye decolourised (Figure 4). By using crude enzymes from culture medium containing RBBR dye, the percentage of RBBR dye decolourisation achieved after an hour was constantly higher as compared to the percentage of RBBR dye decolourised using crude enzyme from culture medium without RBBR dye. This therefore indicated that RBBR dye decolourising enzymes were secreted by isolate MS8 into the culture medium and the decolourisation of the dye by isolate MS8 was by means of extracellular dye degrading enzyme. The production of the dye decolourising enzyme by isolate MS8 was also inducible by RBBR dye.

Comparing the results in Figures 3 and 4, the data shows that the decolourisation of RBBR dye was most certainly due to the activity of Lac enzyme rather than the activity of LiP enzyme. The activity of Lac enzyme is positively correlated to the efficiency of RBBR dye decolourisation. The high percentage of RBBR dye decolourisation when crude enzyme containing high Lac activity was indicative of this as compared with the low percentage of decolourisation when Lac activity was recorded low. The percentage of RBBR dye decolourisation was also the highest on day 12 when RBBR-induced Lac activity peaked. Furthermore, a small amount of RBBR dye was also decolourised, when crude enzyme with low Lac activity but with no detectable LiP activity was used.

### 3.4. Successive Dye Addition and Decolourisation

**Figure 5:**
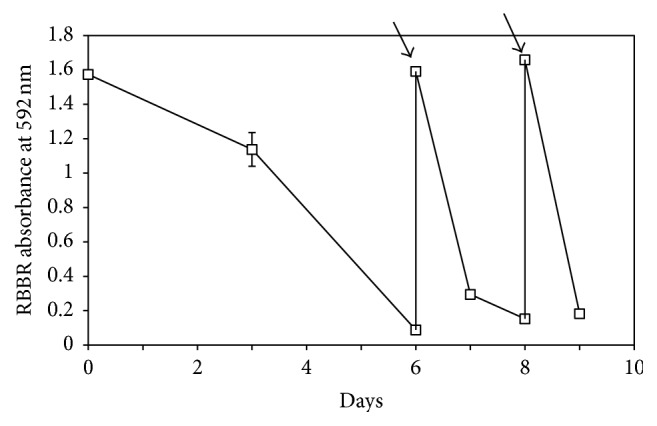
RBBR dye decolourisation by* M. cladophyllus *supplemented twice with fresh RBBR dye. The arrows indicate the addition of fresh RBBR dye (final concentration 200 mg L^−1^) into the fungal culture after dye decolourisation reached more than 80%.

In order to examine the ability of isolate MS8 to continuously decolourise RBBR, fresh RBBR dye was further supplemented to the culture medium when dye decolourisation in the culture reached more than 80%. The results obtained showed not only the decolourisation of all the supplemented dye but also faster decolourisation of the supplemented dye (Figure 5). From the results obtained, 90% decolourisation was achieved in 6 days for the first round of dye added (0.2 g L^−1^) but only 2 days were required to achieve similar level of dye decolourisation in the second round of dye decolourisation. The rate of decolourisation in the third round of dye decolourisation was further increased to just 1 day to achieve around 90% of dye decolourisation. The high Lac activity and decolourisation rate could be seen because the enzyme is continuously produced by isolate MS8 even after day 6 when most of the dye has been decolourised. Hadibarata and coworkers (2012) [5] reported that only 1.5 U L^−1^ of purified laccase was needed to completely decolourise 0.2 g L^−1^ of RBBR. The high Lac enzyme activity recorded in the culture medium on day 6 (136 U L^−1^) must have caused the rapid decolourisation of the freshly supplemented RBBR dye. Laccase enzyme activity continued to increase and peaked on day 12. By the time the second batch of RBBR dye was supplemented on day 8, higher Lac activity was recorded as compared to day 6, therefore sufficiently decolourising the supplemented dye within a much shorter time. The results obtained are in tandem with the work reported by Palmieri and coworkers (2005) [24] on white rot fungus* Pleurotus ostreatus*. Although* P. ostreatus* was reported to decolourise around 80% of RBBR dye in just 3 days, but due to the decrease in Lac activity after day 6, at least 7 days are needed to achieve similar level of dye decolourisation in successive decolourisation cycles.

### 3.5. In Vitro Dye Decolourisation Using Ammonium Sulphate Precipitated Protein

**Figure 6:**
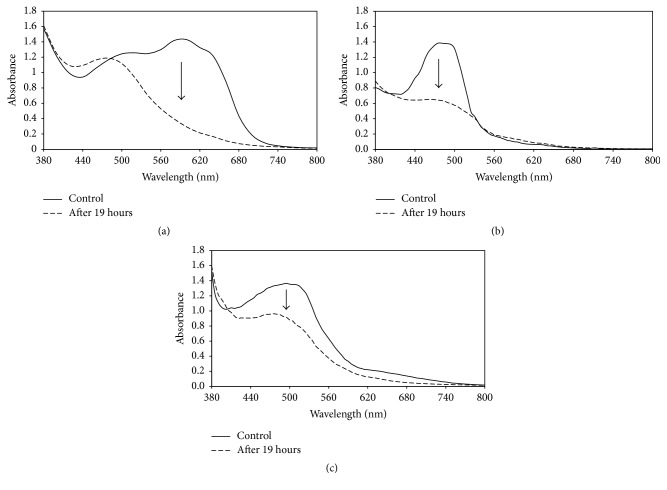
In vitro decolourisation of 0.2 g L^−1^ (a) RBBR dye, (b) Orange G, and (c) Congo red using ammonium sulphate precipitated crude enzyme. Solid line: control spectrum of the tested dye with heat inactivated enzyme. Dashed line represents the spectrum of the tested dye after 19 hours of being treated with enzyme containing 450 U L^−1^ of Lac activity. Arrow indicates the decrease of the spectrum at the maximum absorption wavelength of the respective dyes.

The crude enzyme from the decolourised culture medium was reconcentrated using ammonium sulphate. The protein precipitates that showed RBBR dye decolourisation ability were found to contain only Lac activity with no detectable LiP. We tested the precipitates for in vitro decolourisation of Orange G, a monoazo dye, and Congo red, a diazo dye. The results obtained showed that the protein precipitate containing 450 U L^−1^ of laccase activity was able to decolourise all the tested dye that can be seen in Figures 6(a)–6(c), with the decrease in the spectrum at the maximal absorbance of the dye (arrow) after 19 hours. The reduction and changes to the dyes spectrum further confirmed the degradative decolourisation of the dye by the enzyme produced by isolate MS8. The decolourisation of RBBR dye in 19 hours was the highest reaching up to 76% decolourisation of the initial dye concentration (0.2 g L^−1^). This was then followed by the monoazo dye Orange G with 54% decolourisation and the diazo dye Congo red with 32.9% dye decolourisation.

In comparison to the previously reported dye decolourisation using the live endophytic isolate MS8 [11], dye decolourisation using the enzyme produced by the fungus is much faster and more effective. The dye decolourising enzyme induced by RBBR dye (anthraquinone), apart from the ability to decolourise the inducing dye, was also able to decolourise dyes (azo) which were structurally different from the inducer. This indicated the nonspecificity characteristic of the enzyme. Moreover, the amount of decolourised Orange G dye in just 19 hours using enzyme (54%) was also much higher as compared to the amount decolourised using the live fungus in 16 days (33%) [11]. This therefore highlighted the benefit of using the enzyme produced for the decolourisation of dye which was toxic to the fungus.

### 3.6. Zymograms of Native-PAGE

**Figure 7:**
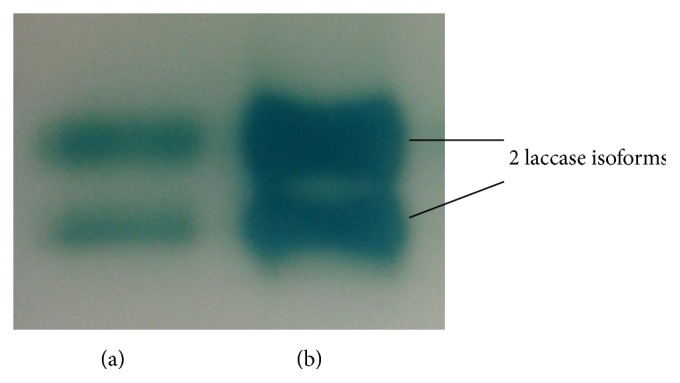
Zymogram of laccase isoforms in (a) RBBR dye decolourised culture medium, 10 *μ*L, and (b) ammonium sulphate precipitated crude protein, 5 *μ*L, after 5 minutes of staining using ABTS.

Lac activity staining using ABTS for the native-PAGE of both the RBBR dye decolourised culture medium and the ammonium sulphate precipitated crude protein caused the formation of two green coloured bands (Figure 7). This shows that, under the culture condition provided, isolate MS8 expressed two laccase isoforms and both of the isoforms were successfully precipitated out of the culture medium and concentrated using ammonium sulphate. The ability of* Marasmius* sp. to produce more than one laccase isoforms has also been reported previously for* Marasmius quercophilus* [15, 16]. Some of these laccase isoforms produced were also reported to have different reactivity towards aromatic compounds. Further purification and characterisation of the two laccase isoforms produced by isolate MS8 are therefore necessary to examine whether both of these laccase isoforms are involved in the decolourisation of synthetic dyes.

Collectively, isolate MS8 showed high potential for industrial application particularly the dye decolourising enzyme for the treatment of wastewater containing dyes. Similar to* Marasmius* sp. reported by Schuckel and coworkers (2011) [18], the endophyte also decolourised synthetic dye by the production of laccase enzyme. Small amount of lignin peroxidase was also produced; however no correlation was found with dye decolourisation. The laccase activity produced by* Marasmius cladophyllus* was induced by RBBR dye and the enzyme activity increases over an extended period of time after RBBR dye decolourisation. Interestingly, the induced laccase activity allowed the fungus to repeatedly decolourise freshly supplemented RBBR dye at a much faster rate [18]. The induced laccase enzyme after decolourising RBBR dye can also be harvested for further dye decolourisation

Large scale production of the enzyme for industrial application however can be very costly. Many researchers therefore have tried to cut down on the cost of enzyme production by using various lignocellulosic materials and agro waste that would induce the enzyme production [30, 31]. With* M. cladophyllus*, it might be feasible to use dye containing wastewater as substrate for the production of dye decolourising enzyme which could simultaneously bring about the decolourisation of the dye wastewater. The treated wastewater will contain the enzyme that could be used for further dye wastewater treatment. Mishra and Kumar (2009) [32] have previously reported on the possibility of using dyeing effluents as an inexpensive culture medium for the production of laccase enzyme by* Coriolus versicolor* and have shown some promising results. The successful application of this technology will not only help in cutting down the cost for enzyme production but also reduce the cost of wastewater treatment.

## 4. Conclusion

The results showed that* M. cladophyllus* was able to decolourise RBBR dye by producing degradative laccase enzyme. Remazol Brilliant Blue R (RBBR) induced the production of laccase enzyme by* M. cladophyllus* and the increased enzyme activity allowed the fungus to repeatedly decolourise additional RBBR dye. Ammonium sulphate precipitated laccase enzyme harvested from decolourised RBBR medium was also able to decolourise azo type dyes. The reusability of the enzyme therefore highlighted its potential use for dye wastewater treatment.
